# Trend, multivariate decomposition and spatial variations of unintended pregnancy among reproductive-age women in Ethiopia: evidence from demographic and health surveys

**DOI:** 10.1186/s41182-022-00440-5

**Published:** 2022-07-19

**Authors:** Daniel Gashaneh Belay, Fantu Mamo Aragaw

**Affiliations:** 1 Department of Human Anatomy, College of Medicine and Health Sciences, University of Gondar, Gondar, Ethiopia; 2 Department of Epidemiology and Biostatistics, Institute of Public Health, College of Medicine and Health Sciences, University of Gondar, Gondar, Ethiopia

**Keywords:** Unintended pregnancy, Reproductive-age women, Trends, Decomposition analysis, Spatial analysis

## Abstract

**Background:**

The magnitude of unintended pregnancy is unacceptably high and more than half of it end up with abortions. This may limit lower and middle-income countries to achieve the sustainable development goal targets of reduction of neonatal and maternal mortalities. Evidence on trends and spatial distribution of unintended pregnancy is limited. Therefore, this study aimed to assess the trend, multivariate decomposition, and spatial variations of unintended pregnancy among reproductive-age women in Ethiopia from 2000 to 2016.

**Methods:**

Ethiopian Demographic and Health Data of 2000 to 2016 were used. A total weighted sample of 30,780 reproductive-age women participated. A multivariate decomposition analysis was employed to identify factors contributing to the change in the rate of unintended pregnancy in Ethiopia for 20 years from (1996 to 2016). The concentration index and graph were used to assess wealth-related inequalities, whereas spatial analysis was done to identify the hotspot of unintended pregnancy in Ethiopia.

**Results:**

The 20-year trend analysis showed that the magnitude of unintended pregnancy among reproductive-age women decreased by 13.19 percentage points (from 39.76% in 2000 to 26.57% in 2016 EDHS). About 84.97% of the overall decrement was due to the difference in coefficient of the variables, whereas the remaining 15.03% was due to the difference in composition of the respondent. The differences in coefficient of the variables were decomposed by living metropolitan cities, having previous terminated pregnancy, and not having exposure to media; whereas, the change due to the composition, was expressed by having a household size of 1–3, living in metropolitan cities, being multipara and grand para, being unmarried and having no terminated pregnancy. Moreover, unintended pregnancies were more clustered in Addis Ababa and disproportionately concentrated in the poor groups.

**Conclusions:**

In Ethiopia, a substantial decrement in unintended pregnancy was observed in the past decade. More than four-fifths of this overall decrement was due to the difference in the coefficient of the variables. There was spatial clustering of unintended pregnancy in Ethiopia. A program intervention is needed for high-risk regions such as Addis Ababa. Health education and media campaign should perform for high-risk women such as those having terminated pregnancy, and professing Islam faith.

**Supplementary Information:**

The online version contains supplementary material available at 10.1186/s41182-022-00440-5.

## Background

Maternal and neonatal mortalities remained unresolved public health problems [1–4]. More than 800 women died every day as a result of pregnancy and childbirth; more than 90% of these deaths were from low-resource countries, and sub-Saharan Africa alone accounts for more than two-thirds of the total [3]. Low resource countries like Ethiopia are off-track in achieving the maternal and neonatal health targets that have been started by the sustainable development goal agenda [2, 5–8].

Unintended pregnancies are unwanted and or mistimed pregnancies at the time of conception [9, 10]. More than half of unintended pregnancies end up with abortions [11, 12]. Unintended pregnancies can reduce the rate of maternal [13–16] and neonatal [17] health services utilization, and worsen maternal health outcomes [10, 18–20]. Even stigma and socio-economic inequalities are common problems of unintended pregnancies [19, 21]. Studies reported that unintended pregnancy is associated with pre-eclampsia, obstetrics bleeding and other maternal complications [10, 22]. The global trend of unintended pregnancy has shown a decreasing trend over time but still, it remains high with significant regional variations [11, 23–26]. Globally, it has been estimated that the magnitude of unintended pregnancy was 44% in 2014 [11]. The magnitude of unintended pregnancy ranges up to more than 50% of the pregnancies in some regions [11]. In lower-income countries, the incidence of unintended pregnancy ranges from 7.2 to 59.6 per 100 person-year of follow-up [26]. Annually more than 14 million unintended pregnancies were reported in sub-Saharan Africa [27] and estimated to be 28% in Ethiopia [28].

Studies showed that socio-demographic and economic characteristics such as age, occupations, marital statuses, residence, wealth status, religion, and women's education have a significant association with unintended pregnancy [9, 27–30]. Moreover, maternal and child health-related factors such as birth order and previous history of neonatal death [30], being multiparous [9, 31], unmet need for family planning [32], experiencing sexual violence [33–35] were also found as predictors of unintended pregnancy.

The Ministry of Health-Ethiopia (MoH) develops a national strategy envisioned to end preventable neonatal deaths by 2035 [8]. But it looks impossible as evidence points out that neonatal mortality is increasing from 29 in the 2016 EDHS to 30 in the 2019 mini EDHS per a thousand live births [7, 36]. It was also planned to reduce neonatal mortality to 11 per thousand live births by 2020 but has not been achieved [7, 8, 36] and needs strong evidence and great commitment to lower it in the coming decades. Maternal health issues are the 1st research priority of sexual and reproductive health as prioritized by the WHO until 2030 [37]. Fighting unintended pregnancy is one way to reducing such high burdens of maternal and neonatal mortalities [12, 16–18, 38, 39]. Hence, assessing the trends and determinants of unintended pregnancy in Ethiopia is very vital for scholars and policy makers.

Therefore, this study aimed to determine the trend and factors associated with the changes in trend in unintended pregnancy and spatial distribution of unintended pregnancy in Ethiopia between 2000 and 2016.

## Methods and materials

### Study design and setting

The study used population-based cross-sectional survey data from EDHS 2000, 2005, 2011, and 2016. Ethiopia is an East African country with 1.1 million square kilometers of coverage and the second-most populous country in Africa with an estimated population of about 115 million in 2021 in 2021 [40]. Administratively, Ethiopia is federally decentralized into eleven regions and two city administrations. Kebele is the lowest administrative unit. Kebles are also subdivided into census enumeration areas (EAs), which are convenient for the implementation of census. The detail of the study design and setting is available elsewhere [41].

### Source and study population

The source population was all reproductive age group women who gave birth 5 years preceding each survey, whereas the study population was women who gave birth in the last 5 years preceding each survey and who lived in the selected enumeration area. Mothers were asked questions about whether the pregnancy was wanted or not for the most last birth [42].

### Sampling technique and sample size

The national survey data were collected using a pre-structured questionnaire. A stratified two-stage cluster sampling technique was employed for all four EDHS surveys using their respective population and housing census as a sampling frame. Child data (KR) were used and extracted the outcome and the independent variables. A nationally representative sample of 30,780 samples (7975 from EDHS 2000, 7308 from EDHS 2005, 7908 from EDHS 2011, and 7590 from EDHS 2016) weighted the number of women who participated (Additional file 1: S1).

### Study variables

The dependent variable for this study was unintended pregnancy which includes pregnancies that are wanted no more (unwanted pregnancy) or wanted later (mistimed pregnancy) [28, 43]; whereas, the independent variables were socio-demographic characteristics, maternal characteristics and behavioral characteristics were also considered (Additional file 2: S2).

### Data processing and analysis

This study employed trend analysis of unintended pregnancy and decomposition of changes in unintended pregnancy using EDHS 2000, 2005, 2011, and 2016 data sets. Data were cleaned, recorded, and analyzed using STATA version 14. As per the recommendation of the survey report, we weighted for the sampling probabilities and non-response using the weighting factor to restore the representativeness of the survey and get reliable statistical estimates before we conduct any statistical analysis.

The trend in unintended pregnancy was analyzed using selected characteristics. The trend was examined separately for the periods 2000–2005, 2006–2011, 2012–2016, 2000–2016. For doing a multivariate decomposition analysis data from EDHS 2000, and 2016 were appended together. The multivariate decomposition analysis decomposes the overall decrease in unintended pregnancy over time into the decrease due to the difference in women’s composition (endowment) across the surveys and the decrease due to the difference in the effect of the characteristics (coefficient) between the surveys. The missing values were clearly defined by the DHS guideline. Missing information on the planning status of birth or the current pregnancy is excluded from the numerators, but included in the denominator. If there were missing values and “don’t know” in the outcome variable (pregnancy wantedness) assumed as not wanted [9]. If the missing value in explanatory variables were greater than 5%, since the DHS survey is a cross-sectional study, the variables were excluded from the further analysis.

### Concentration index and curve

The concentration index is used to quantify the degree and show the direction of socio-economic-related inequality in a health variable. The value of a negative sign indicates a more concentration of unintended pregnancy among the poor, whereas a positive value indicates concentration among the rich.

### Spatial analysis

Spatial analyses were done using ArcGIS version 10.7 and SaTScan version 9.6 software. Global Moran’s I statistic was used to assess whether the spatial distribution of unintended pregnancy was random or non-random. To predict unintended pregnancy in the un-sampled areas based on the values from sampled measurements, the kriging spatial interpolation technique was used. Besides, Getis Ord Gi* statistical hotspot analysis was done to identify the significant areas with high rates and lower rates of unintended pregnancy. Moreover, Bernoulli-based spatial scan statistical analyses were used to detect statistically significant clusters. The primary and secondary clusters were identified and p values were assigned and ranked using their log-likelihood ratio (LLR) test based on the 999 Monte Carlo replications. Areas with high LLR and significant p-value were considered clusters with higher rates of unintended pregnancy.

### Ethical considerations

This study was performed based on the four EDHSs data obtained from the official DHS measure website www.measuredhs.com after permission was obtained via an online request through specifying our analysis objective. The dataset was not shared or passed on to other bodies and has maintained its confidentiality.

## Results

### Characteristics of the study population

The proportion of women who had formal education has decreased from 82.12% to 63.12%, whereas mothers who had attained primary education have increased from 12.56% to 28.32% in 2000 EDHS and 2016 EDHS, respectively. Media exposure among reproductive-age women increased from 27.22% in 2000 to 34.53% in 2016. The proportion of women who are not currently working increased from 42.13% in 2000 to 75.26% in 2016 EDHS (Table 1).

**Table 1:** The characteristics of the study participants of 2000, 2005, 2011, and 2016 Ethiopian Demographic and Health survey

Variables	Categories	Weighted frequency (%) EDHS 2000	Weighted frequency (%) EDHS 2005	Weighted frequency (%) EDHS 2011	Weighted frequency (%) EDHS 2016
Maternal age (in years)	15–19	473 (5.92)	440 (6.02)	402 (5.08)	339 (4.47)
	20–24	1728 (21.66)	1474 (20.16)	1609 (20.34)	1466 (19.30)
	25–29	2026 (25.40)	1961 (26.83)	2383 (30.13)	2166 (28.53)
	30–34	1490 (18.76)	1428 (19.54)	1490 (18.83)	1662 (21.89)
	35–39	1220 (15.29)	1138 (15.57)	1239 (15.67)	1207 (15.89)
	40–44	707 (8.85)	579 (7.91)	572 (7.22)	547 (7.20)
	45–49	329 (4.12)	290 (3.96)	216 (2.73)	270 (2.73)
Residence	Urban	908 (11.38)	634 (8.67)	1189 (15.00)	969 (12.77)
	Rural	7067 (88.62)	6674 (91.33)	6719 (84.98)	6621 (87.21)
Current marital Status	Currently not married	785 (9.84)	535 (7.32)	723 (9.14)	482 (6.34)
	Currently married	7190 (90.16)	6773 (92.68)	7186 (90.86)	7109 (93.66)
Household size	1–3	1112 (13.94)	910 (12.44)	1080 (13.65)	1033 (13.61)
	4–6	4047 (50.74)	3644 (49.86)	4057 (51.30)	3889 (51.23)
	> = 7	2817 (35.31)	2754 (37.76)	2772 (35.05)	2669 (35.16)
Religion	Orthodox	4054 (50.83)	3263 (44.64)	3328 (42.07)	2883 (37.97)
	Protestant	1232 (15.45)	1464 (19.21)	2563 (32.41)	1652 (21.76)
	Muslim	2338 (29.31)	2383 (32.60)	250 (3.16)	2825 (37.21)
	Other	352 (4.41)	260 (3.55)	1769 (22.36)	233 (3.06)
Region	Small peripherals	273 (3.42)	449 (6.14)	399 (5.04)	442 (5.81)
	Large centrals	7511 (94.18)	6691 (91.57)	7271 (91.94)	6900 (90.96)
	Metropolitans	191 (2.40)	168 (2.30)	239 (3.01)	250 (3.28)
Media exposure	No	5798 (72.28)	4564 (62.46)	3171 (40.10)	4970 (65.47)
	Yes	2169 (27.22)	2743 (37.54)	4737 (59.90)	2621 (34.53)
Maternal working status	No	3359 (42.13)	5500 (75.26)	5140 (64.94)	5418 (71.38)
	Yes	4614 (57.87)	1809 (24.47)	2769 (35.01)	2173 (28.62)
Maternal educational level	No formal education	6549 (82.12)	5735 (78.47)	5271 (66.64)	4792 (63.12)
	Primary	1002 (12.56)	1206 (16.49)	2271 (28.71)	2150 (28.32)
	Secondary and above	425 (5.32)	368 (5.04)	368 (4.65)	650 (8.55)
Household head	Male	6758 (84.75)	6420 (87.85)	6912 (89.42)	6474 (85.29)
	Female	1217 (15.25)	888 (12.15)	1297 (16.40)	1117 (14.71)
Parity	Prime	1363 (17.08)	1191 (16.29)	1400 (17.69)	1435 (18.90)
	Multi	3262 (40.90)	3026 (41.40)	3465 (43.81)	3190 (42.02)
	Grand	3350 (42.02)	3092 (42.31)	3045 (38.50)	2966 (39.08)
Ever had terminated pregnancy	No	6735 (84.46)	6689 (91.53)	7072 (89.42)	6910 (91.04)
	Yes	1240 (15.54)	619 (8.47)	837 (10.58)	680 (8.96)
Knowledge of ovulatory cycle	Don’t know	1760 (22.06)	2387 (32.66)	2002 (25.31)	1254 (16.51)
	Know	6215 (77.94)	4921 (67.34)	5907 (74.69)	6337 (83.49)

### The trend of unintended pregnancy in Ethiopia

**Fig. 1:**
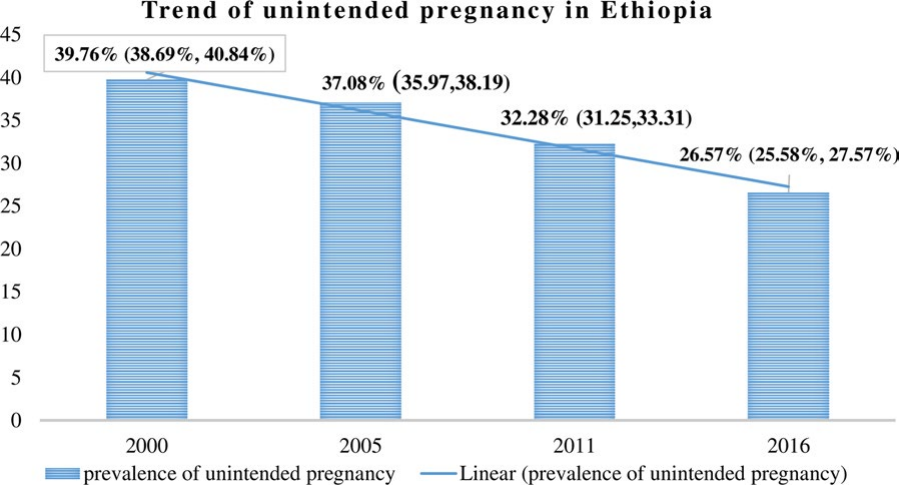
Trends of unintended pregnancy among reproductive-age women in Ethiopia

The overall trend of unintended pregnancy among reproductive-age women has decreased from 39.76% (95% CI 38.69%, 40.84%) in 2000 EDHS, to 26.57% (95% CI 25.58%, 27.57%) in 2016 EDHS (Fig. 1).

The difference in confidence interval does not overlap in either of each phase (2000–2005, 2005–2011, 2011–2016, and 2000–2016), which indicates that the change in the proportion of unintended pregnancy was significant in each phase. In women with secondary and higher education, urban residence, and living in metropolitan cities, unintended pregnancy declined by 33.84, 26.98, and 21.11 percentage points in the entire study period between 2000 and 2016, respectively (Table 2).

**Table 2:** Trends of unintended pregnancy among reproductive-age women by characteristics in 2000, 2005, 2011, and 2016 Ethiopia Demographic and Health Surveys

Variables	EDHS 2000	EDHS 2005		EDHS 2011		EDHS 2016	Point difference in unintended pregnancy					
							Phase 1 2005–2000	Phase 1 2011–2005	Phase 2 2016–2011		Phase 1 2016–2000	
Maternal age												
15–19	43.02	36.88		33.72		27.25	− 6.14	− 3.16	− 6.47		− 15.77	
20–24	38.10	35.92		30.41		21.31	− 2.18	− 5.51	− 9.1		− 16.79	
25–29	37.02	33.94		30.83		24.77	− 3.08	− 3.11	− 6.06		− 12.25	
30–34	38.04	35.19		30.40		26.44	− 2.85	− 4.79	− 3.96		− 3.96	
35–39	42.28	40.17		35.35		30.13	− 2.11	− 4.82	− 5.22		− 12.15	
40–44	47.45	45.77		37.32		36.47	− 1.68	− 8.45	− 0.85		− 10.98	
45–49	39.76	44.29		41.32		35.55	4.53	− 2.97	− 5.77		− 4.21	
Marital status												
Not married	53.45	47.09		42.41	36.52		− 6.36	− 4.68		− 5.89	− 16.93	
Married	38.27	36.29		31.26	25.89		− 1.98	− 5.03		− 5.37	− 12.38	
Residence												
Rural	37.9	38.76		33.57	25.90		0.86	− 5.19		− 7.67	− − 12	
Urban	53.64	36.92		32.05	26.66		− 16.72	− 4.84		-5.39	− 26.98	
Maternal education												
No education	37.52	35.75		31.51	26.46		− 1.77	− 4.06		− 5.05		− 11.44
Primary education	47.22	42.92		34.38	27.89		− 4.3	− 8.54		− − 6.49		− 19.24
Secondary and above	56.82	38.60		30.33	22.98		− 18.22	− 8.27		− 7.35		− 33.84
Region												
Small Peripherals	22.21	11.96		14.39	7.31		− 10.25	2.43		− 7.08		− 14.9
Large centrals	40.18	38.72		33.25	27.77		− 1.46	− 5.47		− 10.48		− 12.41
Metropolitans	48.55	38.50		32.43	27.44		10.05	− 6.07		− 4.99		− 21.11
Media exposure												
No	36.62	33.56	29.32		26.41		− 3.06	− 4.24		− 2.91		− 10.21
Yes	42.09	42.93	34.25		26.86		0.84	− 8.68		− 7.39		− 15.23
Parity												
Primiparous	33.68	32.44	26.83		19.69		− 1.24	− 5.61		− 7.14		− 13.99
Multiparous	37.74	32.28	30.67		24.22		− 5.46	− 1.61		− 6.45		− 13.52
Grand multiparous	44.21	43.56	36.61		32.42		− 0.65	− 6.95		− 4.19		− 11.79
Knowledge of ovulatory cycle												
Don’t know	38.15	36.59	29.36		26.24		− 1.56	− 7.23		− 3.12		− 11.91
Know	40.22	37.32	33.26		26.63		− 2.9	− 4.06		− 6.63		− 13.59
Prevalence 95% CI	39.76 (38.69, 40.84)	37.08 (35.97, 38.19)	32.28 (31.25, 33.31)		26.57 (25.58, 27.57)		(− 2.72, − 2.65)	(− 4.72, − 4.88)		(− 5.67, − 5.74)		(− 13.11, -13.27)

### Multivariate decomposition analysis

The overall multivariate decomposition analysis revealed that about 84.97% of the overall decrease in unintended pregnancies who gave birth in the last 5 years was due to the difference in coefficient (difference in the effect of characteristics) across the surveys, whereas the remaining 15.03% of the overall decrease in unintended pregnancy was due to the difference in composition of the respondent (endowment) across the surveys (Table 3).

**Table 3:** The overall decomposition analysis of the decrease in unintended pregnancy among reproductive-age women in Ethiopia, 2000 to 2016

Unintended pregnancy	Coefficient (95%CI)	Percentage
Endowment	− 0.019 (− 0.034, − 0.005)	15.03
Coefficient	− 0.511 (− 0.138, − 0.862)	84.97
Residual	− 0.132 (− 0.153, − 0.111)	

Among the change due to composition (endowment), household size of 1–3, having terminated pregnancy, living in the large central and metropolitan cities, multipara and grand para women, and unmarried women were significantly contributed to the decrease in unintended pregnancy over 16 years (from 2000 to 2016).

The increase in composition of woman who have a house hold size of 1–3 [β = 0.0004, 95% CI 0.0001, 0.0006] and multipara [*β* = 0.0008, 95% CI 0.0002, 0.001] had a counteracting effect on the decrement of unintended pregnancy by 0.3% and 0.6%, respectively. A decrease in composition of woman who ever had terminated pregnancy [*β* = − 0.003, 95% CI − 0.007, − 0.0004], grand multiparous [*β* = − 0.004, 95% CI − 0.006, − 0.002] and live in large central region [*β* = − 0.109, 95% CI − 0.12, − 0.009] contribute for the decrement of unintended pregnancy by 2.88%, 3.4% and 8.2%; whereas, the increase in composition of women who live in metropolitans cities [*β* = 0.002, 95% CI 0.0022, 0.003] contribute for the increment of unintended pregnancy by 2.1% (Table 4).

**Table 4:** The detailed decomposition analysis of the change in unintended pregnancy among reproductive-age women in Ethiopia from 2000 to 2016

Variable	Difference due to characteristics (*E*)		Difference due to coefficient (*C*)	
	Coefficient	Percent	Coefficient	Percent
Maternal age				
15–19	− 0.0011 (− 0.0029, 0.0008)	0.794	− 0.0055 (− 0.161, 0.0049)	4.18
20–24	0.0002 (− 0.0023, 0.0027)	− 0.161	− 0.0196 (− 0.0503, 0.0110)	14.86
25–29	− 0.0008 (− 0.0037, 0.0022)	0.575	− 0.0101 (− 0.0433, 0.0229)	7.69
30–34	− 0.0016 (− 0.0044, 0.0013)	1.2	− 0.0068 (− 0.0301, 0.0163)	5.20
35–39	− 0.0002 (− 0.0008, 0.0003)	0.178	− 0.0062 (− 0.025, 0.0128)	− 4.66
40–44	− 0.0001 (− 0.0017, 0.0015)	0.059	− 0.0027 (− 0.0144, 0.0089)	2.07
45–49	−	−	−	−
Maternal education				
No education	0.0043 (− 0.0091, 0.0178)	− 3.27	0.0619 (− 0.0256, 0.1496)	− 46.89
Primary education	0.0031 (− 0.0071, 0.0133)	− 2.32	0.0070 (− 0.0060, 0.0200)	− 5.31
Secondary and above	−	−	−	-
Residence				
Urban	–	–	–	–
Rural	0.0006 (− 0.0004, 0.0015)	− 0.42	0.0426 (− 0.0360, 0.1213)	− 32.27
Region				
Small peripherals	–	–	–	–
Large centrals	− 0.0109 (− 0.0124, − 0.0094)**	8.26	0.172 (0.1158, 0.2286)**	− 130.3
Metropolitans	0.0028 (0.0022, 0.0033)**	− 2.106	0.005 (0.0032, 0.0078)**	− 4.20
Marital status				
Not married	− 0.0042 (− 0.0065, − 0.0018)	3.14	− 0.0027 (− 0.0113, 0.0059)	2.04
Married	–	–	–	–
Occupational status				
Working	− 0.005 (− 0.0155, 0.0047)	4.11	0.0014 (− 0.0436, 0.04465)	− 1.08
Not working	–	–	–	–
Religion				
Orthodox	-–	–	–	–
Protestant	− 0.0015 (− 0.004, 0.001)	1.11	0.0057 (− 0.0034, 0.0150)	− 4.386
Muslim	0.0009 (− 0.0021, 0.0038)	− 0.664	0.0169 (0.0011, 0.0327)*	− 12.81
Other	− 0.0011 (− 0.0004, 0.0026)	− 0.834	− 0.0002 (− 0.0064, 0.0061)	0.15
Household head				
Male	0.00002 (− 0.00022, 0.00017)	0.017	− 0.0503 (− 0.1069, 0.0063)	38.074
Female	–	–	–	–
Household size				
1–3	0.0004 (0.0001, 0.0006)**	− 0.3	− 0.0052 (− 0.0177, 0.0073)	3.94
4–6	− 0.0001 (− 0.0001,0.00001)	0.10	0.0020 (− 0.0257, 0.0297)	− 1.52
> = 7	-–	–	–	–
Had terminated pregnancy				
No	-	–	–	
Yes	− 0.003 (− 0.007− ,0.0004)*	2.82	0.0163 (0.0058, 0.0267)**	− 12.33
Media exposure				
No	− 0.0003 (− 0.003, 0.0023)	0.21	0.0506 (0.0123, 0.0891)**	− 38.33
Yes	–	–	–	–
Parity				
Primiparous	–	–	–	–
Multiparous	0.0008 (0.00022, 0.0015)**	− 0.64	− 0.0083 (− 0.0415, 0.0249)	6.286
Grand multiparous	− 0.0045 (− 0.00678, − 0.0022)*	3.406	− 0.0136 (− 0.0576, 0.0304)	10.324
Knowledge of ovulatory cycle				
Don’t know	–	–	–	–
Know	− 0.0003 (− 0.0027, 0.002	0.26	0.0014 (− 0.0436, 0.0465)	− 1.08

^*^Significance at *P*-value < 0.05, **significance at *P*-value < 0.01

After controlling the role of compositional changes, 84.97% of the decrease in unintended pregnancy over 16 years was attributed to the difference in coefficients (the effects of characteristics) (Table 3). This was explained by significantly contributed factors such as living in large central and metropolitan cities, having previous terminated pregnancy, and media exposure About 12.8% and 12.33% of the change of unintended pregnancy over 16 years were due to the difference in the effect Muslim religion follower women [*β* = 0.016, 95% CI 0.001, 0.32], and having previous terminated pregnancy [*β* = 0.016, 95% CI 0.005, 0.026].

An increase in the effect of not exposed to media [*β* = 0.05, 95% CI 0.012, 0.089], living in large central region [*β* = 0.172, 95% CI 0.115, 0.228] and metropolitans cities [*β* = 0.005, 95% CI 0.003, 0.007] contributed for the increment of unintended pregnancy by 38.33%, 130.30% and 4.23%, respectively (Table 4).

### Wealth-related inequality in unintended pregnancy

**Fig. 2:**
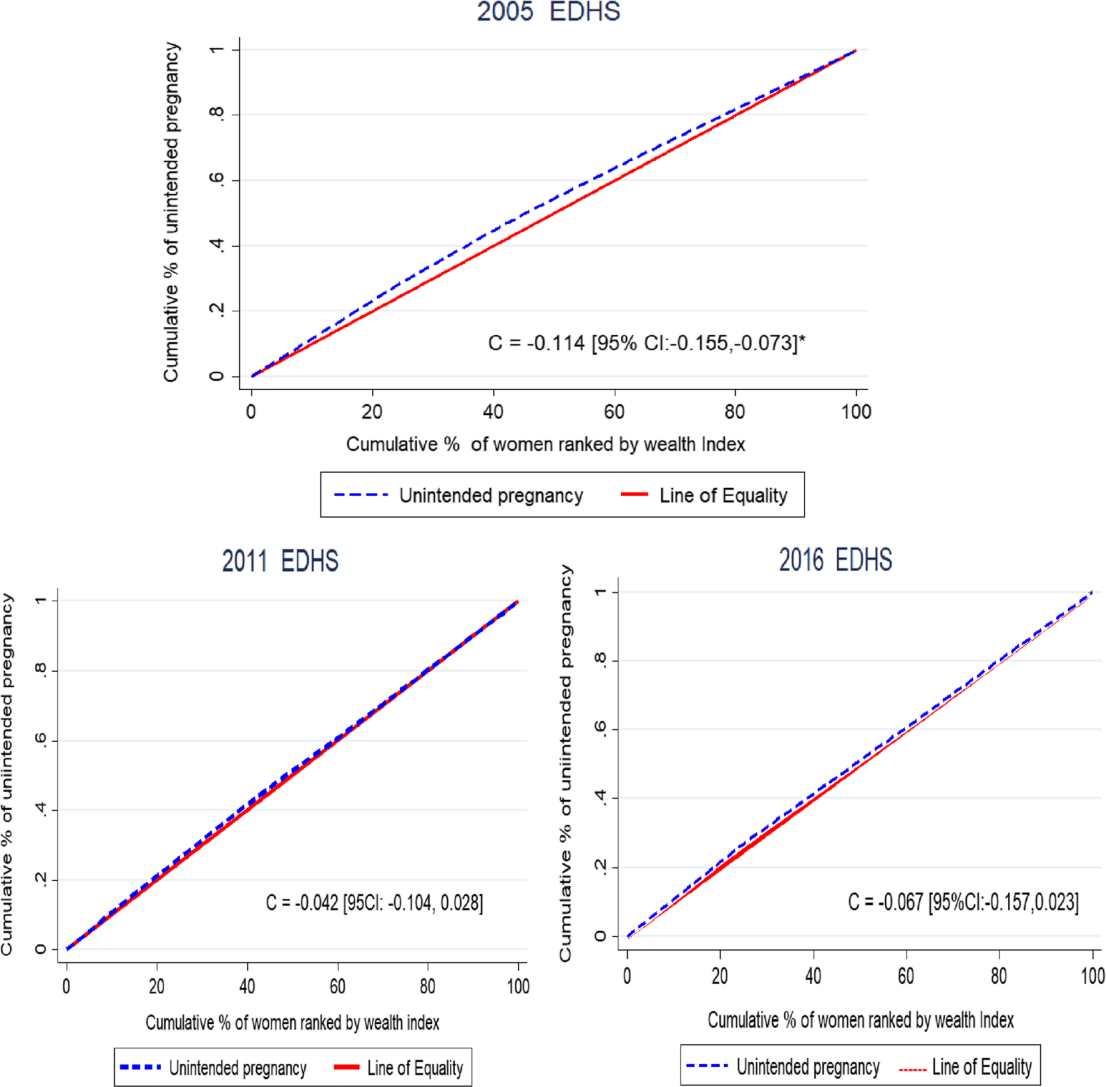
The concentration graph shows wealth-related inequalities of unintended pregnancy in Ethiopia

In this study, the concentration index and curve were assessed for the last three EDHS (2005, 2011, and 2016). Since it has no wealth index variables in EDHS 2000, wealth related to equality was not assessed. The Wagstaff-normalized concentration index (*C*) analyses of the wealth-related inequality of unintended pregnancy were significant only in EDHS 2005 and showed that the pro-poor distribution of unintended pregnancy with [*C* = − 0.114; 95% CI − 0.115, − 0.073] and the graph of unintended pregnancy is above the line of equality. This shows that unintended pregnancy among reproductive-age women was disproportionately concentrated in the poor groups (pro-poor distribution) (Fig. 2).

### Spatial distribution, autocorrelation and kriging interpolation of unintended pregnancy among reproductive-age women, EDHS 2000–2016

**Fig. 3:**
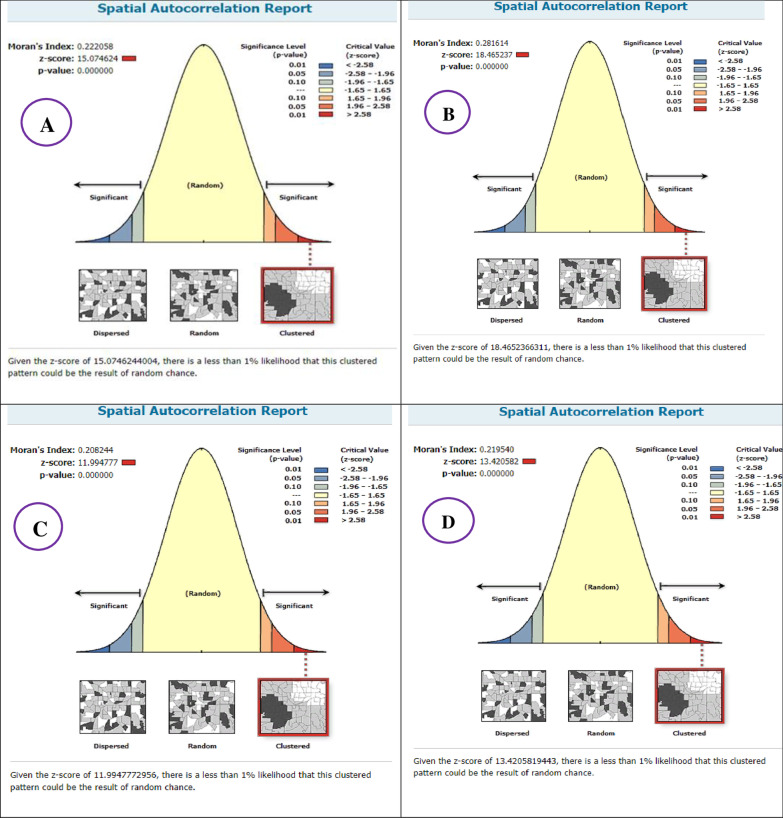
Spatial autocorrelation analysis of unintended pregnancy among reproductive-age women in Ethiopia, EDHS 2000 (**A**), 2005 (**B**), 2011 (**C**), and 2016 (**D**)

Spatial distribution of unintended pregnancy among reproductive-age women showed significant spatial variation across the country over time. In all EDHS, the spatial distribution of unintended pregnancy among reproductive-age women was found to be non-random with Global Moran’s I value 0.22, 0.28, 0.20, and 0.21 (*P* < 0.001), respectively. All have clustered patterns of distribution (Fig. 3).

The spatial interpolation of the predicted proportion of women with unintended pregnancy among reproductive-age women over the area increases from green to red-colored in the following figures. The prevalence of high-risk areas predicted unintended pregnancy decreased progressively from 65% in EDHS 2000 to 44.22% in EDHS 2016.

**Fig. 4:**
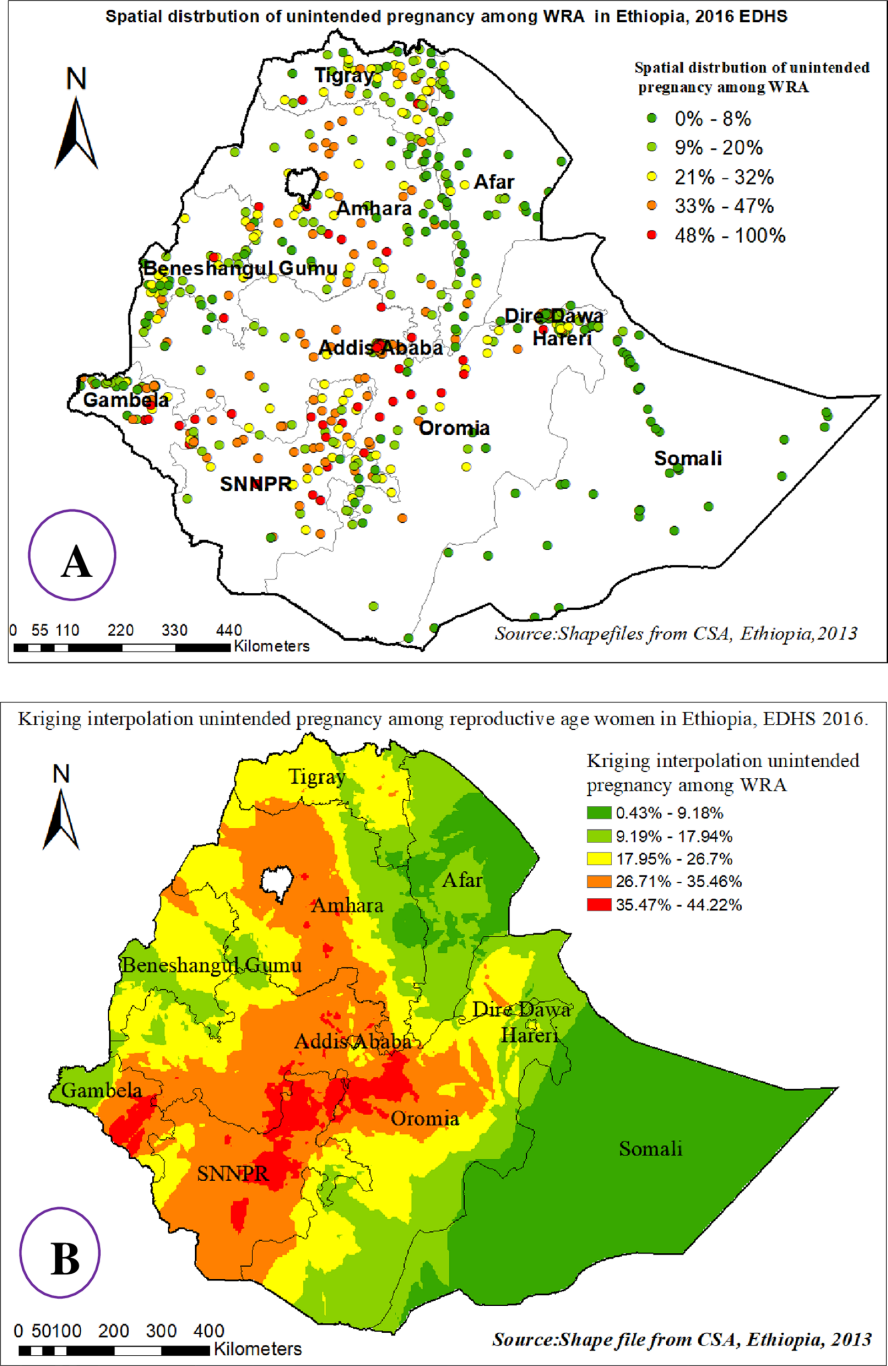
Spatial distribution (**A**) and kriging interpolation (**B**) of unintended pregnancy among reproductive-age women in Ethiopia, 2016 EDHS

Based on EDHS 2000 the highest unintended pregnancy was predicted in the North Shewa zone and Eastern parts of the Amhara, Diredawa, and Addis Ababa regions whereas the predicted relatively low unintended pregnancies were located in the Somali, and Afar regions (Additional file 3: S3). In 2005 EDHS, Kriging interpolation revealed that the predicted highest prevalence of unintended pregnancy was found in almost the entire Oromia and SNNP region. In contrast, low predicted unintended pregnancies were detected in Tigray, Afar, Somali, and Western parts of the Gambella region (Additional file 4: S4). From EDHS 2011 data, Kriging interpolation predicted that the entire Oromia, Western Amhara, entire Benishangul-Gumuz, and Eastern parts of Gambella regions contained the highest unintended pregnancy, while Tigray, Afar, and Somali, regions contained relatively low unintended pregnancy problems (Additional file 5: S5). In the recent EDHS 2016, data Kriging interpolation predicted that the North and South Gondar zone and East Gojam zone of Amhara, Addis Ababa, almost the entire SNNP, and Gambella regions predicted the highest prevalence of unintended pregnancy, whereas Afar, Somali, eastern Amhara, and southeast Oromia regions predicted relatively low unintended pregnancy (Fig. 4).

### Hot spot (Getis-Ord Gi*) and SaTccan analysis of unintended pregnancy among reproductive-age women Ethiopia, 2000–2016 EDHS

**Fig. 5:**
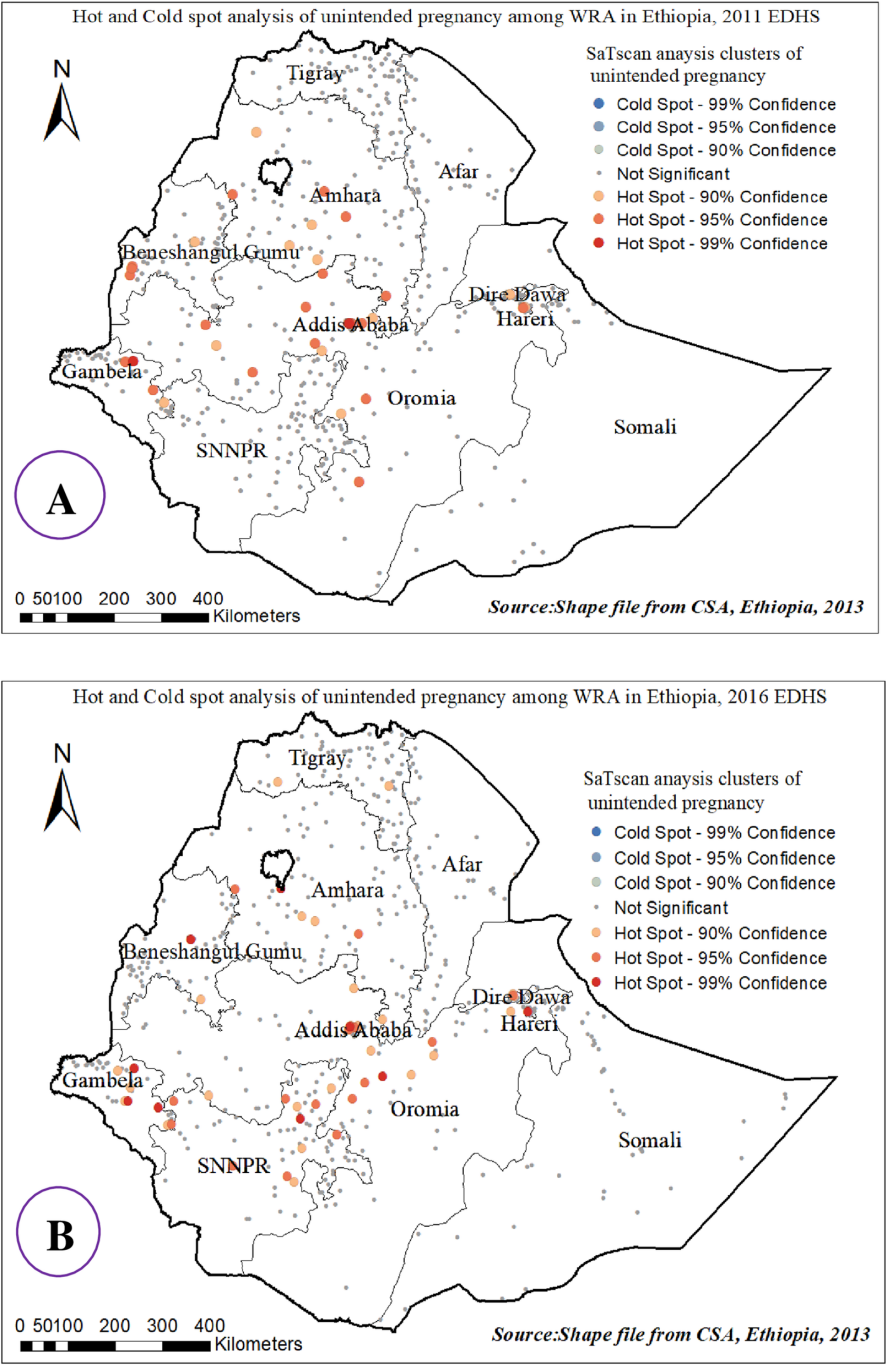
Hot and cold spot areas of unintended pregnancy among reproductive-age women in Ethiopia, 2011 EDHS(**A**) and 2016 EDHS (**B**)

As indicated in the following figure, the red color indicates the more intense clustering of a high (hot spot) proportion of unintended pregnancy among reproductive-age women. Overall a high proportion of unintended pregnancies was identified in Amhara, Addis Ababa Oromia, Diredewa, Gambella, and the SNNP region of Ethiopia; whereas Tigray, Afar, and Somali, regions were fewer risk areas consistently over time (Additional file 6: S6 and Fig. 5).

In SaTscan analysis, most likely primary and secondary clusters of unintended pregnancy among women of reproductive age (WRA) were identified. A total of 266, 247, 272, and 269 most likely primary clusters were identified in 2000, 2005, 2011, and 2016 EDHS, respectively. Moreover, in 2011 EDHS 48 secondary clusters were identified. It showed that women within the spatial window had a 1.5, 2, 1.8, and 1.9 times higher risk of unintended pregnancy than women outside the window in 2000, 2005, 2011, and 2016 EDHS, respectively [RR, *P*-value ≤ 0.001] (Additional file 7: S7).

**Fig. 6:**
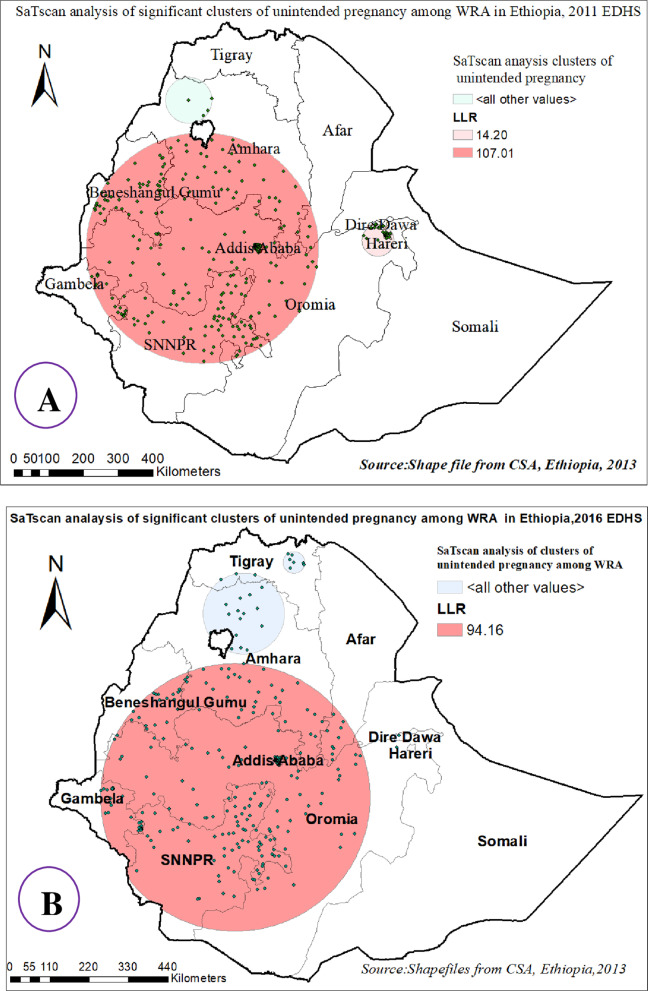
SaTscan analysis of unintended pregnancy among reproductive-age women Ethiopia, 2011 EDHS (**A**) and 2016 EDHS (**B**)

Overall the SaTScan analysis revealed that south Amhara, Addis Ababa, and Oromia and SNNP regions were persistently at higher risk of unintended pregnancy across the four surveys (Additional file 8: S8 and Fig. 6).

## Discussion

This study aimed to determine the trend, determinant factors for the changes, and spatial distribution of unintended pregnancy in Ethiopia over the past 16 years.

In this study, the magnitude of unintended pregnancy among reproductive-age women has decreased from 39.76 point prevalence in 2000 to 26.57 point prevalence in 2016 EDHS. This is higher than a study done in India in 2015 (14.47%) [46] and evidence from six south Asian countries (19.1%) [47]. But this study is lower than nearly a study in the United States which shows half (49%) of pregnancies were unintended in 2006 [48]. This was explained by pregnancy intention in developing countries being influenced by socio-cultural, environmental, individual, and health service-related factors [28]. In addition, in developed countries childbearing becomes unwanted and comes later in life [47, 48]. Moreover, in Ethiopia, there is an increase in the availability and accessibility of different family planning services in both urban and rural areas [49]. Women's autonomy and reproductive health-care-seeking behavior also improved [50].

The result of this study revealed that the contribution of behavior (coefficient) changes was more important than that of composition (endowments) changes to the decline of unintended pregnancy in the past 16 years. This might be due to the that there was a small change in the structural composition of unintended pregnant women in the surveys.

In this study, a decrease in the composition of grand multiparous, a woman who has given birth 5 or more times women contributes to the decrement of unintended pregnancy, and also an increase in the composition of multiparous women contributes to the increment of unintended pregnancy. This is in line with the study conducted in Iran [51] and a study conducted in Ethiopia [52, 53]. This may be related to the number of children a woman would have, the more the number of the child, the more it becomes unintended pregnancy. In addition, there was low utilization of contraceptives in multiparous (24.02%) and grandparous (17.20%) women as compared to primi in our data. Moreover, an increased family size of one–three members had a negative contribution to the decrement of unintended pregnancy over time. This finding is contrary to a study done in Ethiopia [45]. The possible justification might be those who have large family sizes may be due to high unplanned births.

Our finding showed that the decrease in the composition of unmarried women over time contributed to the decrement in unintended pregnancy. This is similar to other studies conducted in Ethiopia [52, 54–56]. This may be due to that, in Ethiopia, having children without a married (single mother) is not accepted by most of the community. Therefore, unmarried women might have more experience with unintended pregnancy than married women. Moreover, the study showed that married women used contraceptives more likely than unmarried ones.

Our finding showed that a decrease in the composition of women who ever had terminated the pregnancy had contributed to the decrement of unintended pregnancy and also an increase in the effect of having terminated pregnancy significantly contributes to the increment of unintended pregnancy. The possible justification might be, in our data, there is low utilization of contraceptives in a woman who ever had terminated pregnancy (16.64%) as compared to those who haven’t (20.78%).

In our study, an increase in the effect of being a Muslim religion follower had increased unintended pregnancy. This finding is in line with other studies conducted in Ethiopia [9, 45] and. This may be due to low contraceptive utilization among Muslims (14.62%) than orthodox Christians (24.40%) in our study. This is supported by other similar studies [9, 45]. Moreover, Muslim women’s activities were more restricted than some other religions, and also they were likely to accept pregnancy as “given by Allah” [47].

Moreover, in this study increase, the effect of no media exposure contributes to the increment of unintended pregnancy [56]. The reason for this may be women with access to mass media have better information about contraceptives and are more likely to use family planning services [47].

The concentration index analyses of the wealth-related inequality of unintended pregnancy showed that unintended pregnancy is more distributed in poor women (pro-poor distribution). This is supported by studies that show that stigma and socio-economic inequalities are common problems of unintended pregnancies [19, 21]. Most unintended pregnancies occurred in the poorest women due to raped or commercial sex workers [46]. Moreover, in our study, there is a decreased proportion of women with formal education and an increased proportion of unemployed women. Eventually, these women have poor household wealth status and intern results in the occurrences of unintended pregnancies.

In this study, an increase in the composition of a woman living in the metropolitan cities (Addis Ababa, Harari, and Dire Dawa), and a decrease in the composition of a woman living in the large central region (Tigray, Amhara, Oromia, SNNPR) were contributed for the increment of unintended pregnancy over the past sixteen years. This is also supported by the spatial distribution results which suggest that the highest unintended pregnancy were identified and predicted in Diredewa and Addis Ababa and some parts of the Amhara regions. This is in line with previous studies conducted in Ethiopia [45, 57]. A study in six south Asian countries [47] and India [46] also showed that unintended pregnancies have spatial clusters. This might be due to, the expected high prevalence of commercial sex workers in metropolitan and large central regions than in other regions of Ethiopia and if pregnancy might have occurred it will be more likely to be unintended.

This study had several strengths, the study was based on nationally representative large datasets, and thus, it has adequate statistical power and can be generalized to all reproductive-age women in the study setting. However, this study is not free from limitations. Since the data were collected cross-sectional by self–reported interviews, it would be prone to recall and social desirability bias. The drawback of the secondary nature of data was inevitable. We miss important variables like behavioral and other family planning service quality, access, and service utilization-related parameters.

## Conclusions

Even though the magnitude of unintended pregnancy was high, a substantial decrement was observed in the past decade. More than four-fifths of the decrement was attributed to the change in behavioral factors over time. The differences in effects of living in the large central and metropolitan cities, having previous terminated pregnancy, not having media exposure, and being a woman who is Muslim were the factors that significantly contributed to the decrease in unintended pregnancy over time. While the change in the composition of women who have terminated pregnancy, women having a household size of 1–3, unmarried women, multiparous women, and grandparous, and women who live in large central and metropolitan cities were significantly contributed to the change in unintended pregnancy over 16 years. The spatial distribution, of unintended pregnancy, was non-random in Ethiopia in which the primary clusters’ of unintended pregnancy were identified in central Addis Ababa. The Ethiopian Minister of Health better use this work as baseline information to take action to further decrease unintended pregnancy among women. It is better to focus on high-risk regions to further reduce the burden of unintended pregnancy and perform a media campaign to increase women’s awareness of maternal health, especially those who are disadvantaged.

## Supplementary Information

**Additional file 1. **Sample size and sampling procedures in a study of unintended pregnancy among reproductive-age women in Ethiopia.

**Additional file 2. **Table which shows the independent variables in the study of trend, multivariate decomposition, and spatial variations of unintended pregnancy among reproductive-age women in Ethiopia.

**Additional file 3. **Spatial distribution (A) and kriging interpolation (B) of unintended pregnancy among reproductive-age women in Ethiopia, 2000 EDHS.

**Additional file 4. **Spatial distribution (A) and kriging interpolation (B) of unintended pregnancy among reproductive-age women in Ethiopia, 2005 EDHS.

**Additional file 5. **Spatial distribution (A) and kriging interpolation (B) of unintended pregnancy among reproductive-age women in Ethiopia, 2011 EDHS.

**Additional file 6. **Hot and cold spot areas of unintended pregnancy among reproductive-age women in Ethiopia, 2000 EDHS (A) and 2005 EDHS (B).

**Additional file 7. **Table shows that significant spatial primary and secondary clusters analysis result of unintended pregnancy among reproductive-age women in Ethiopia, 2000, 2005, 2011, and 2016 EDHS.

**Additional file 8. **StaTCan analysis of unintended pregnancy among reproductive-age women Ethiopia, 2000 EDHS (A) and 2005 EDHS (B).

## Data Availability

Data are available online and can be accessed from www.measuredhs.com.

## Abbreviations

CI: Confidence Interval
CSA: Central Statistical Agency
CDHS: Cambodia Demographic Health Survey
DHS: Demographic and Health Survey
EDHS: Ethiopian Demographic and Health Survey
EPHI: Ethiopian Public Health Institute
PSU: Primary Sampling Unit
Mvdcmp: Multivariate decomposition analysis
WHO: World Health Organization
