# Supplementary figures and images for: Trend, multivariate decomposition and spatial variations of unintended pregnancy among reproductive-age women in Ethiopia: evidence from demographic and health surveys
Source: Trop Med Health. 2022 Jul 19;50:47. doi: 10.1186/s41182-022-00440-5 (PMC9295486; doi:10.1186/s41182-022-00440-5)

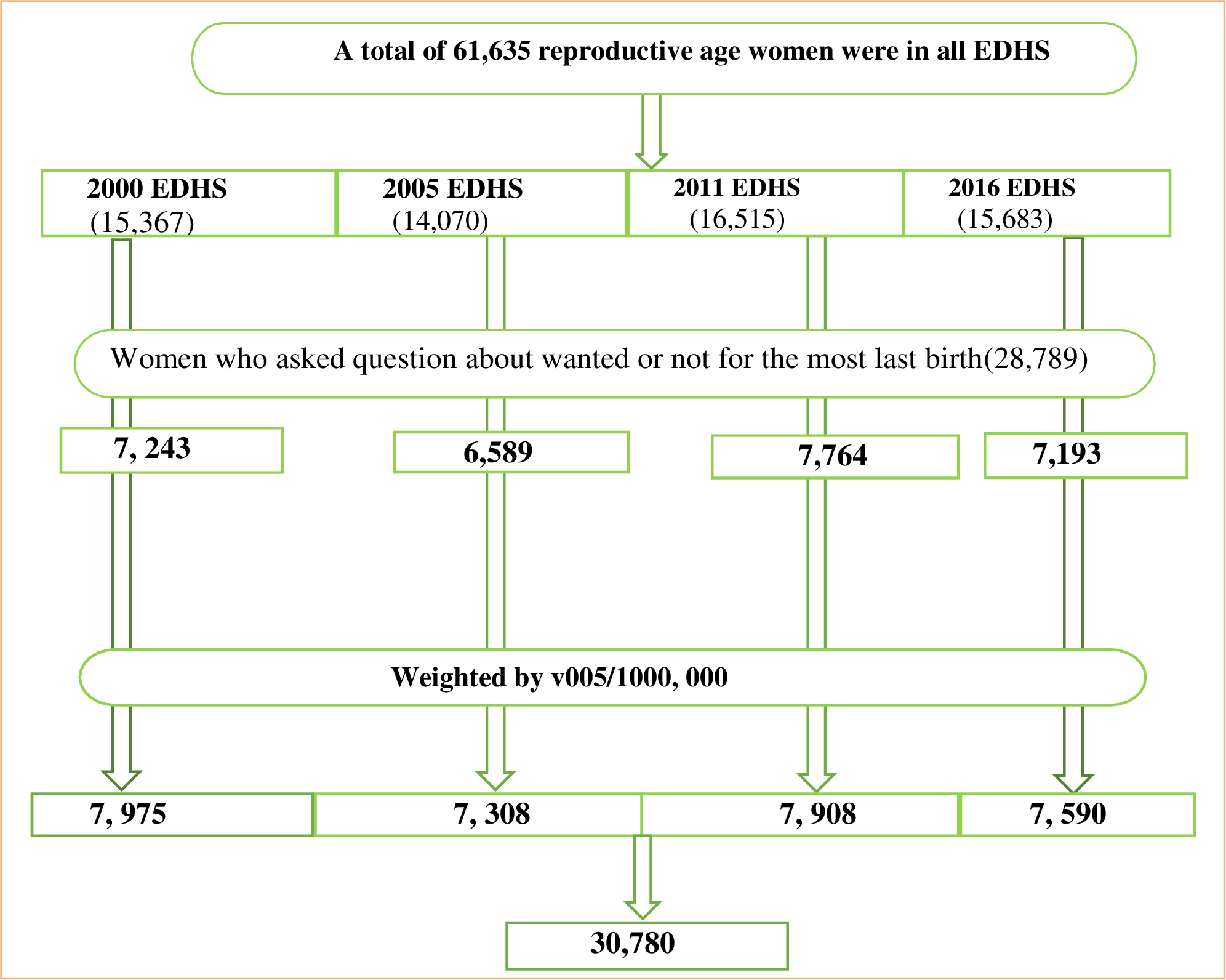

Supplement: Supplementary file 1 — Additional file 1. Sample size and sampling procedures in a study of unintended pregnancy among reproductive-age women in Ethiopia. [file 41182_2022_440_MOESM1_ESM.tif]

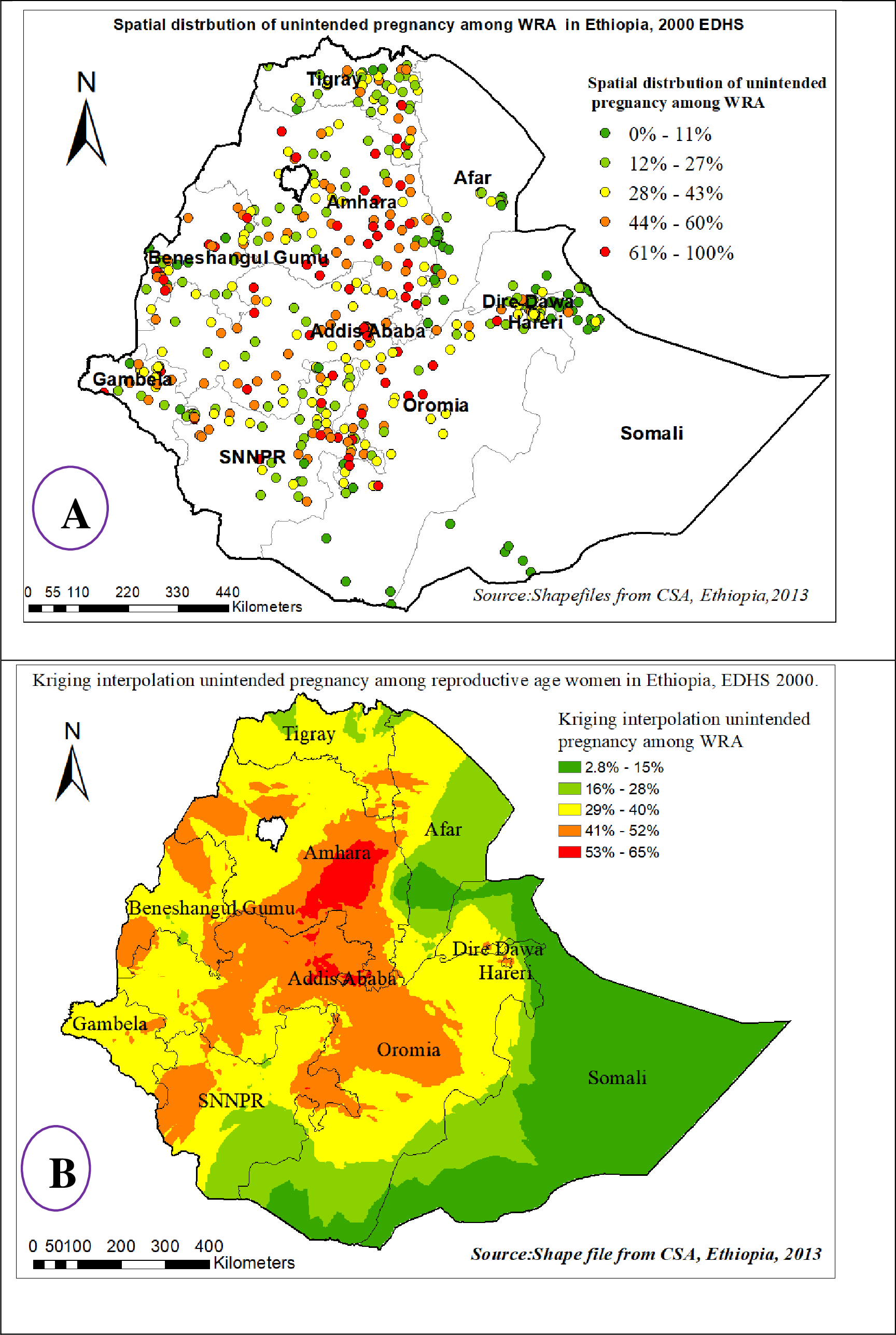

Supplement: Supplementary file 3 — Additional file 3. Spatial distribution (A) and kriging interpolation (B) of unintended pregnancy among reproductive-age women in Ethiopia, 2000 EDHS. [file 41182_2022_440_MOESM3_ESM.tif]

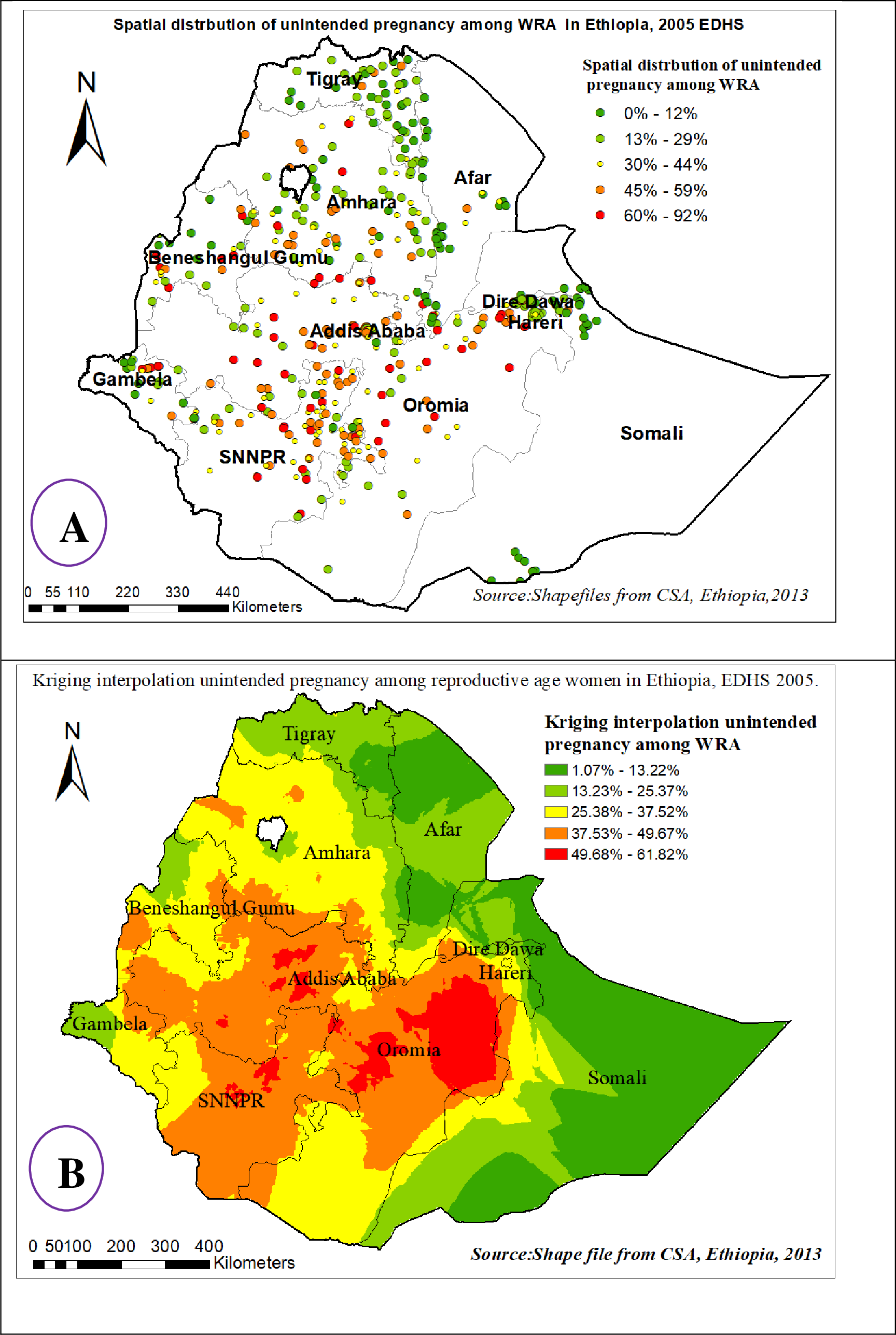

Supplement: Supplementary file 4 — Additional file 4. Spatial distribution (A) and kriging interpolation (B) of unintended pregnancy among reproductive-age women in Ethiopia, 2005 EDHS. [file 41182_2022_440_MOESM4_ESM.tif]

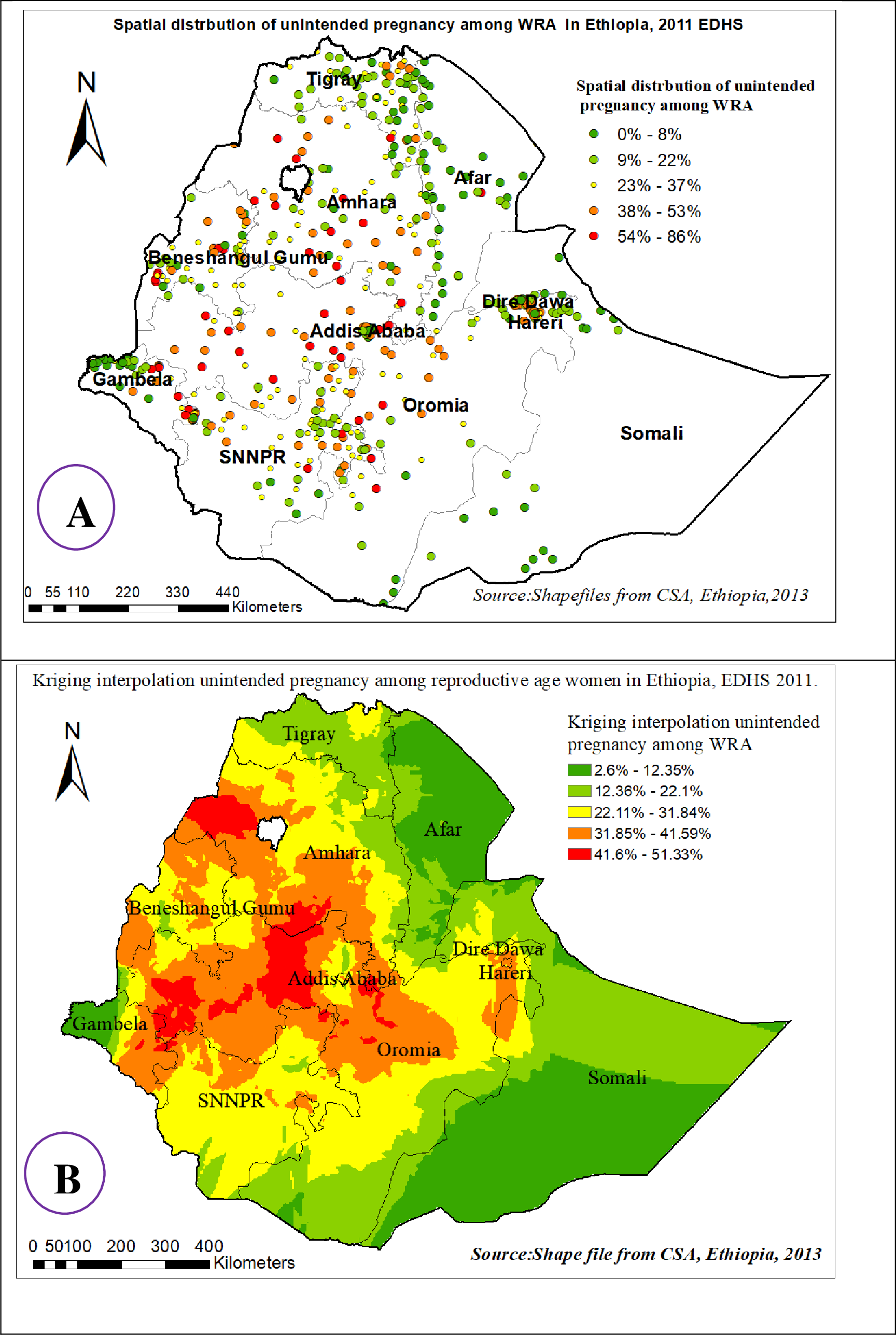

Supplement: Supplementary file 5 — Additional file 5. Spatial distribution (A) and kriging interpolation (B) of unintended pregnancy among reproductive-age women in Ethiopia, 2011 EDHS. [file 41182_2022_440_MOESM5_ESM.tif]

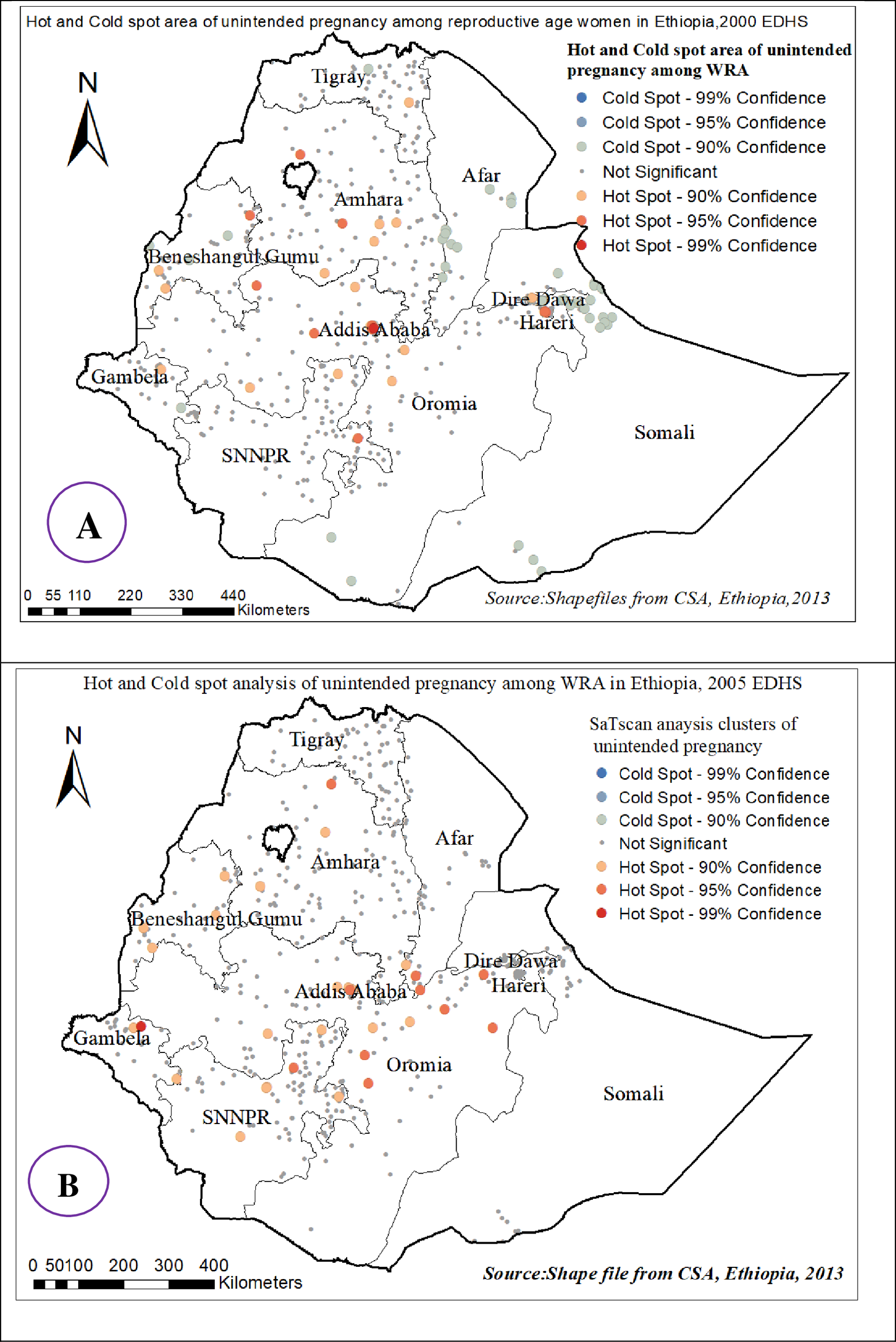

Supplement: Supplementary file 6 — Additional file 6. Hot and cold spot areas of unintended pregnancy among reproductive-age women in Ethiopia, 2000 EDHS (A) and 2005 EDHS (B). [file 41182_2022_440_MOESM6_ESM.tif]

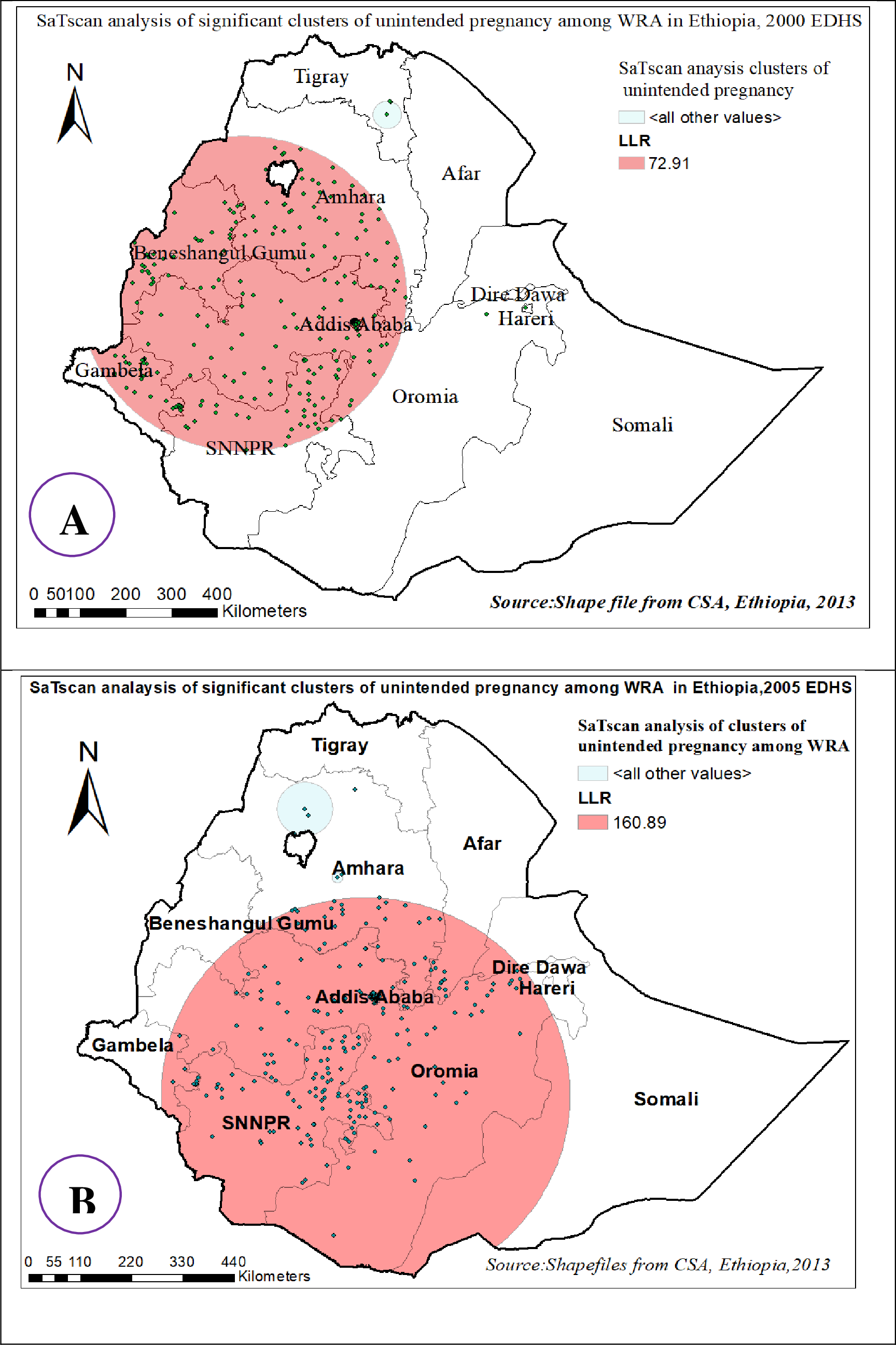

Supplement: Supplementary file 8 — Additional file 8. StaTCan analysis of unintended pregnancy among reproductive-age women Ethiopia, 2000 EDHS (A) and 2005 EDHS (B). [file 41182_2022_440_MOESM8_ESM.tif]
