# Supplementary material for: Trend, multivariate decomposition and spatial variations of unintended pregnancy among reproductive-age women in Ethiopia: evidence from demographic and health surveys
Source: Trop Med Health. 2022 Jul 19;50:47. doi: 10.1186/s41182-022-00440-5 (PMC9295486; doi:10.1186/s41182-022-00440-5)
Supplement: Supplementary file 2 — Additional file 2. Table which shows the independent variables in the study of trend, multivariate decomposition, and spatial variations of unintended pregnancy among reproductive-age women in Ethiopia. [file 41182_2022_440_MOESM2_ESM.pdf]

S2: Table which shows the independent variables in the study of trend, multivariate decomposition, and spatial variations of unintended pregnancy among reproductive-age women in Ethiopia.

**Variables**	**Measurements**
Maternal age	The age of the mother/caregiver is categorized as 15-19, 20-24, 25-29, 30-34, 35-39, 40-44 and 45-49.
Education level	Educational attainment is categorized as No education, primary, secondary, and above educational status.
Marital status	The marital status of the mothers is categorized as married or not married.
Occupation of women	The occupation of women is categorized as working and not working.
Family size	Categorized as 1-3, 4-6, .7, and above.
Religion	Religions are classified as Orthodox, Protestant, Muslim, and other.
Media exposure	A composite variable was obtained by combining whether a respondent reads newspaper/magazine, listens to the radio, and watches television with a value of "0" if women were not exposed to at least one of the three media, and "1" if a woman has access/exposure to at least one of the three media [44].
Wealth index	The datasets contained a wealth index that was created using principal components analysis coded as poorest, poorer, middle, richer, and richest in the DHS data set. For this study, we recorded it in three categories poor (including poorer and poorest), middle and rich (includes richer and richest).
Head of household	The sex of the household head is categorized as males and females.
Ever had terminated pregnancy	The terminated pregnancy history can be classified as "yes" and "no".
Parity	Parity is classified as prim parous, multiparous, and grand multiparous when having five and more children.
Knowledge of ovulatory cycle	Knowledge of the ovulatory cycle is classified as "know" and 'don't know.
Residency	Urban or rural based on where the household lives.
Region	The region is recoded as, the three Metropolis' (which include Addis Ababa, Harari, and Dire Dawa), large central (Tigray, Amhara, Oromia, SNNPR), and "small peripherals" (which include Afar, Benshangul-Gumuz, Gambelia, and Somali) [45].
