# Supplementary material for: Trend, multivariate decomposition and spatial variations of unintended pregnancy among reproductive-age women in Ethiopia: evidence from demographic and health surveys
Source: Trop Med Health. 2022 Jul 19;50:47. doi: 10.1186/s41182-022-00440-5 (PMC9295486; doi:10.1186/s41182-022-00440-5)
Supplement: Supplementary file 7 — Additional file 7. Table shows that significant spatial primary and secondary clusters analysis result of unintended pregnancy among reproductive-age women in Ethiopia, 2000, 2005, 2011, and 2016 EDHS. [file 41182_2022_440_MOESM7_ESM.pdf]

Table 6. SaTScan analysis result of unintended pregnancy among reproductive-age women in Ethiopia, 2000,2005,2011, and 2016 EDHS.

**EDHS**	**EDHS 2000**	**EDHS 2005**	**EDHS 2011**		**EDHS 2016**
Cluster	Primary	Primary	Primary	Secondary	Primary
Number of clusters	1 (266)	1 (247)	1 (272)	2(48)	1 (269)
Coordinate /Radius	9.594732 N, 36.535397 E/ 354.78 km	7.031568 N, 38.601410 E / 437.93 km	8.975787 N, 37.322113 E / 332.97 km	9.169772 N, 4 1.909794 E / 45.93 km	8.053772 N, 37.694584 E / 375.50 km
Population	3586	3197	3457	696	3101
Cases	1514	1348	1169	240	872
RR	1.47	1.96	1.78	1.38	1.91
LRR	72.910	160.892	107.047	14.201	94.164
P-value	P = 0.001	P < 0.0001	P < 0.0001	P = 0.0005	P < 0.0001
